# On the Treatment of the Insane

**Published:** 1842-01

**Authors:** John Conolly, Chas. Aug. Tulk

**Affiliations:** Resident Physician of the County Asylum at Hanwell; Chairman


### On the Treatment of the Insane. By John Conolly, m.d., Resident Physician of the County Asylum at Hanwell.

[We have more than once called the attention of our readers to the admirable system of treating the insane, followed by the resident physician of the Middlesex County Asylum. The establishment and carrying out of this system, as it has been done at Hanwell, while it reflects eternal honour on Dr. Conolly, has been already, and must continue more and more to be, of inestimable benefit to the insane throughout the whole civilized world. We feel it, therefore, to be a duty imposed upon us as journalists, to lend our utmost aid to the promulgation of the plans of treatment advocated and enforced by Dr. Conolly; and with this view, we extract from the "Third Report of the Resident Physician," dated October 1st, 1841, all that relates to *bodily restraints, seclusion, general treatment,* and *religious services.* These admirable and conclusive observations we recommend to the earnest consideration of all our readers, and especially to all those gentlemen who are in any way connected with the insane in lunatic houses.]

"Disuse of Bodily Restraints.—In the First Report presented to the visiting justices by the present resident physician (in October 1839), the general views by which he was guided in the commencement of his duties at the asylum were briefly stated. In his Second Report (in October 1840), those views were more fully explained, particularly such of them as appeared to him to be least clearly understood in other asylums. During the past year the same general plan of management has been steadily acted upon, and, with the continual help of the visiting justices, all the arrangements of the building and establishment have been gradually adapted, or are in the course of adaptation to their fulfilment.

"It is unnecessary for the resident physician to dilate on such principles as are now admitted into every well conducted asylum for insane persons. To ascertain, if possible, the remote or immediate origin of the malady in every case, and to derive from this enquiry some rules of successful treatment, to endeavour to gain and preserve the confidence of each patient, to create or to maintain a character of kindness and tranquillity throughout the asylum, together with a vigilant superintendence; to forbid the exercise of violence, threats, or deception; to classify the patients in the manner most likely to promote their recovery; to be careful of their diet and clothing; to occupy and to amuse them; to secure them cheerfulness or content by day, and comfortable rest by night; to consider all their weaknesses and infirmities, and to pay a general regard to whatever may act favorably on the mind and body; to abstract every cause of hurtful excitement, and to encourage every hopeful symptom of mental recovery; these are the objects professed by every rational physician to whom the insane are entrusted, and the time is fast approaching in which lunatic asylums will only be distinguished from one another by the skill and perseverance with which the infinite means of accomplishing these objects are varied and applied.

"Their application at Hanwell must always derive additional importance from the great extent of the asylum, and the large proportion of incurable patients at all times contained in it. Whilst the relative number of cures, and even the mortality in any asylum will be found to depend in a great measure on the rules regulating the admission and discharge of patients, the relative amount of comfort and happiness in asylums should be equal in all, or dependent only on the forms of disease respectively contained in them. In the acute and early stages of mania and melancholy, sensibility may be oppressed, and the patient may appear indifferent to places, persons, and all varieties of treatment. Experience shows that this oppression, even in the acute stage, is more apparent than real; and that deep impressions are made, good or bad, as the treatment may vary, and not afterwards to be effaced. But in the chronic form of mental disorder, which comprehends every conceivable variety of mental wreck that may survive the storms of the first attack, the patient's well-doing is entirely dependent on his general management. To those, unfortunately many in number, for whom no hope of cure remains, the asylum is the earthly home; the only friends their distress has left them are under the roof with them, and they will only leave it for the grave. For them, therefore, the daily and habitual life takes its colour from the general principles which animate those at the head of the establishment in which they are placed. It may be rendered abject and wretched by indifference or by ignorance; and it may be diversified with a large variety of happiness by enlightened benevolence. If computation fails to show this, it is plainly appreciable by observation, the result being the greatest degree of general tranquillity, safety, and order, with the smallest possible display of direct control, and an entire absence of severity. The great aim of the superintendent of a lunatic asylum must ever be to effect these objects by a simplicity of means essential to their constant application, and by a concurrence of innumerable indirect influences, which, systematically brought to bear on those committed to his charge, render direct command and visible authority rare and rarely required.

"More than two years have now been completed since the resident physician began to report to the visiting justices the gradual disuse of mechanical modes of restraint, and the substitution of a more efficient superintendence by means of a greater number of attendants of intelligence and respectability; his motives, as then stated, were the apparent inefficacy of mechanical restraint as a means of preventing accidents and mischief; its irritating effects on the violent, the alarm it occasioned to the timid, and its tendency to debase those to whom it was applied, and to create incurable habits of uncleanliness. The resident physician gratefully remembers the consideration these representations received from the visiting justices in the autumn of 1839; and the kindness and constancy with which his early efforts to abolish a mode of treatment so objectionable were regarded by them, in the midst of difficulties which he is now happy to be able to refer to as entirely overcome.

"In October, 1840, after a year's experience, made in circumstances some of which were singularly unfavorable to the success of the trial, he felt himself justified in announcing its probable success. Another year has now passed, and the experiment has for a part of this year been conducted by a united household. From the moment of this union of purpose among the officers and attendants, the obstacles besetting the experiment have diminished, accidents and assaults have become less frequent, and the general tranquillity and order of the asylum have increased. However unpleasant the recollection of the state of the asylum may be at the time of the commencement of the change of system, it affords the important knowledge that confusion among the patients is the concomitant of unfaithfulness in those on whose exertions the superintendent must depend, and the preservation of discipline easy when those whose office it is to preserve it are zealous and sincere.

"Every successive month appears to add to these encouraging effects, the attendants daily acquiring a clearer appreciation of a system from which violence of every kind is excluded. Its advantages are thus more effectually secured to patients newly admitted; and the general results strengthen the conviction that by abolishing restraints many other evils, considered inseparable from lunatic asylums, are at the same time swept away.

"In the course of the year, several patients have been admitted in restraints, and many more marked with restraints imposed before admission. The management of all these cases has proved perfectly practicable without restraints. In general, the patients have seemed very sensible of the benefits of their emancipation; and in some instances in which patients so marked, marked indeed for life, have recovered, they have delivered a consistent and impressive relation of their former treatment, and expressed their belief that it was extremely injurious to them. In one case, that of a fine young man, of excellent temper (J. E.), both ancles and both wrists were ulcerated, and he informed us that this was done by handcuffs and leg-locks, worn before admission, and the whole body so emaciated that he seemed to be sinking fast into the grave. For a short time this patient was occasionally troublesome, and he was now and then secluded; but he gradually became quiet in his behaviour, he grew stout, was employed in the store-room, and in four months went away quite well, and with the good wishes of all who knew him. This young man repeatedly said that he owed his life to his removal to Hanwell.

"In another case (H. L.), a young man, was admitted whose back was covered with ulcers, and he was apparently paralysed in his lower extremities. He can now walk about; and there is too much reason to believe that the account given by his relatives is correct, and that his ulcers, and the temporary privation of the use of his lower limbs, were occasioned by his having been long fastened down in bed, and consequently, as always happens when patients are so restrained, often lying on wet bedding.

"Very recently a female patient (M. R.) was admitted, who was supposed to have lost the power of walking in consequence of paralysis; she is also gradually recovering, and walks about. Her friends report that she was always fastened down before she came to us. She proves to be perfectly harmless; and, being an elderly woman, the use of her lower limbs would probably have soon been irrecoverably lost if she had not been removed.

"Great, and sometimes insuperable, difficulty is experienced in attempting to gain the confidence of patients who have been treated with austerity or subjected to restraints before admission. They can scarcely be convinced that fair words conceal no guile, or that they will be allowed to sleep unbound. One patient (C. C.), a poor girl of 20, held out her hand a long time after going to bed for the first time at Hanwell, and could with difficulty be persuaded by the nurse that it would not be strapped to the side of the bed. She said she was accustomed to have one hand and both feet fastened at night. Her general demeanour proved to be remarkably quiet; but now and then she was excited, and benefited by leeches, baths, and even by temporary seclusion. She now looks so stout and cheerful as scarcely to be recognized as the depressed creature who came to us in August, but she is still in a state requiring occasional medical treatment. On the same day a stout florid young Irishwoman was admitted, very irascible, and loudly complaining of having been violently treated. There were marks of manacles on her wrists. In a few days her behaviour much improved. She soon became occupied in the laundry, and then a valuable helper in the kitchen of one of the officers, and in less than two months she left the asylum perfectly well.

"Cases of a somewhat similar description are too numerous for particular detail; and they are only alluded to as illustrating, by their subsequent course, the indiscriminate abuse of restraints of which the patients have been the unfortunate subjects. The importance of admitting patients of the poorer class as soon as possible into the asylum, when they become affected with insanity, cannot be overstated. Young persons are brought to us who have been a few weeks maniacal, and, in consequence of the alarm or ignorance of those about them, have beeu locked up by the police, forcibly tied down in workhouses, exposed to severity and insult, and have had handcuffs put upon them, and been eventually sent to the asylum much exasperated by all that had passed. This was remarkably exemplified in a delicate young woman of 18, (M. S.), whose recovery conmenced from the date of her admission, although she was both maniacal and disposed to suicide when she was first received, and who has left the asylum quite recovered. The wild gestures and frantic aspect of this young creature began to be lost when she had been two days in the wards. She became remarkable for mildness and amiability of conduct, but retained a strong rccollection of the manner in which she had been treated before she was sent to us.

"To those who have opportunities of observing the extraordinary changes wrought in the most violent recent cases, by continual patience and kindness, it cannot but appear probable that some among the older patients, who remain immoveably sullen or morose, might have been benefited at an earlier period, if they had not been treated roughly and without consideration.

"A prolonged maniacal attack is not unfrequently characterized by continual activity and a most ingenious disposition to mischief. When restraints were employed, these restless and troublesome patients were very frequent subjects of it. It prevented the necessity for the almost continual watching required, and which was too irksome to be borne by an attendant who could at once be relieved from his care by putting the patient's hands in a leather muff, or locking his ancles together. The inconveniences then created fell chiefly on the patient, and many such patients were by degrees allowed to be either in constant or in very frequent restraint, always greatly to their detriment, and sometimes to their entire ruin. The patients now alluded to are seldom violent; they are easily amused, and when amused are as playful as children, but they are irritable, and become uncertain in their temper under the annoyance of mechanical restraints. In the commencement of the attack, there is often evident bodily disorder, and it is one of the most serious of the evils attendant on bodily restraints that patients so treated do not receive that share of medical attention which they require. A strong illustration of this kind of case presented itself to the resident physician within the last three months.

"A male patient (R. E.) was admitted on the 23d of June, and reported to be 'dangerous to those about him.' It was avowed that in the asylum from whence he came he had been kept almost constantly in instrumental restraint for three months. He laboured under some religious and other delusions, was almost always talking, and somewhat restless in his habits, so that although emaciated, and suffering from disorder of the stomach and bowels, it was not practicable to keep him among the quiet and feeble patients in the infirmary. He was placed on that account in a ward assigned to more troublesome patients, but he was of course never subjected to restraint; and, although fidgety and always in action, he occasioned so little solicitude to attendants trained to habitual vigilance, that he was never once even put in seclusion. A red and coated tongue, a voracious appetite, a disposition to swallow grass or gravel, frequent vomiting, irritable bowels, and a rapid pulse, afforded plainer indications for medical treatment than are usually met with in the insane, and clearly pointed out the case as one requiring remedial means rather than physical coercion. The state of the digestive organs became the subject of especial attention, and dictated a careful medicinal and dietetic treatment, under which this poor man has gradually recovered health and strength of body, and is fast advancing to the recovery of his mental faculties. In September he began to work in the garden, he is placed in one of the quietest wards, he writes affectionate and rational letters to his wife, and he lately partook of the sacrament in the chapel at his own request.

"It is never possible for a physician to say of any case that it was treated on erroneous principles at a period anterior to that when his own observation of it commenced; but it is certainly difficult to conceive the necessity for mechanical restraints, and particularly for continual restraints, in a case of this description.

"T. F., a poor Irish labourer, was admitted in February last, looking wild and half starved. He had worn a strait-waistcoat, he said, in the workhouse from whence he came, and had been tied down in his bed. His mental disorder seemed the mere result of misery and starvation. The common comforts of the asylum composed him, he instantly began to recover, and is now perfectly well. He was placed in no kind of restraint or seclusion, but lodged in the infirmary and treated as a sick man. The excuse for dwelling on the value of attentions which every physician would consider both simple and obvious, is, that where restraints are at the constant command of the attendants, their ready imposition so entirely alters the aspect of the case that it scarcely gains the physician's attention at all.

"A female patient (S. P.) was admitted in January, labouring under complicated bodily disease, of which she died. The delirium which attended her malady had been thought indicative, before her admission, of the necessity of putting her into a strait-waistcoat. She had formerly been a nurse in this asylum, and told us that she helped those about her to put on the waistcoat, as they did not understand how to do it, a circumstance at least showing how little necessity there was for such an application. The frequent imposition of a strait-waistcoat on a submissive patient by a single attendant was one of the circumstances which originally awakened the attention of the resident physician to its habitual abuse.

"Incidents exemplifying the success attending the persevering application of diversified means to all kinds of cases, instead of restraint, are too frequent and too numerous to be reported. Thus (R. S. S.), labouring under acute mania, occasioned much trouble at night by his restlessness; various medicinal applications were tried without success, and his room and dress were so arranged, that his restless nights could be followed by no bad consequences on his health, but he was never fastened to his bed. At length it occurred to the house-surgeon of the male side, that malt liquor sometimes proved an agreeable sedative. A bottle of Scotch ale was given to the patient at night with the most satisfactory effects, and continued for some weeks; the quantity was then gradually reduced, but its omission for a single night was still followed by bad effects. During this time, the patient who had been reduced to the state of a skeleton, and was generally excessively noisy, has become fat and in all respects greatly improved. He has been able to attend at chapel, and he sometimes plays the flute whilst other patients dance, and seems in good humour with everybody about him.

"Less striking cases were, however, among the more unhappy victims of restraints; restless, helpless creatures, seldom speaking, and seeming almost wholly stupid, and scarcely exciting attention. In a case of this kind (E. H.), a poor feeble man, could not always be induced to lie down on his bed; he sometimes remained a great part of the night, or the whole of it, standing at the door as if ready to come out. He slept much by day, seemed well nourished, seldom spoke, and never complained, but his ancles began to swell. Continued attention from the night-attendants and the keeper of the infirmary, together with a new kind of bed, in the course of adoption, instead of straw beds, (and presently to be described,) at length had the effect of habituating this patient to lie in his bed at night; and he may now often be seen by day no longer sleeping or stupid or silent, but cleaning the knives and forks, polishing the fire-irons, and pointing to and telling of the result of his exertions, with much apparent satisfaction, and he appears in perfectly good health. It is unquestionable, that if this poor man had been fastened in his bed by hand or foot, he would have become less and less capable of exertion, and that he would have soon entirely lost his health, and would also have remained in restraint every night until he died. The resident physician cannot forget having more than once discovered that dying patients were not released from restraints. Even in the restlessness of death, their feet were strapped or chained to the bedstead, and an order to liberate them seemed to occasion surprise.

"The resident physician feels himself called upon to speak of these subjects with less reserve than he has previously maintained, being assured that his efforts, and those of the visiting justices, to abolish useless coercion, would have met with no dissentient voice if the extent of its abuse had not been too considerably veiled. The certainty of abuse creeping on the use of coercion, whenever it is permitted, is an argument against it which nothing can overcome.

"Although the resident physician has abstained from taking part in the controversy carried on without intermission in various publications since the appearance of his last Report, considering it his duty to content himself with the opportunity of making, in another Annual Report, a faithful and unimpassioned statement to the visiting justices, he begs to say that he does not refrain from particular allusion to the animadversions made on his former statements with any feeling of disrespect towards those who conscientiously differed from him. For any personal observations, or unjust censure cast upon him, he has more than found a compensation in the approbation of his efforts expressed by the visiting justices, by whom alone they were fully known. He has also continually had the satisfaction to perceive that the members of the medical profession, although engaging on different sides of the question as to the abolition of restraints, have honoured him with the same courtesy and kindness which he has experienced from them on all former occasions. He willingly ascribes the manifestation of any contrary feeling in any other quarter to a want of opportunity of seeing the questioned system in actual operation, and to a forgetfulness that, whether the system be right or wrong, its professed object is to avoid unnecessary cruelty.

"Several hypothetical, and some real cases of complicated difficulty have been adduced as proving that there were many forms of mental disorder wholly unmanageable without restraints. Having never presumed to say that no possible case could ever occur in which a resort to restraints might be found convenient, the resident physician has perused these cases carefully. Of all of them it may be observed, that they would have been more complete if an attempt to treat them without restraint had formed any part of the narrative. Apparently such attempts were not made, and the impossibility of doing so was only assumed. It has also been with surprise that he has not yet met with one related case, with one imaginary combination of danger and difficulty, of which he does not know, from actual observation, that the management is practicable, and the evils are avoidable, without having recourse to such measures.

"It is moreover an important fact, that in the only asylums in England in which attempts are known to have been made to abolish restraints, the experiment is reported to have been successful, as in the asylums of Lincoln, where the experiment was first tried, Melton (Suffolk), Northampton, and Lancaster, the last the largest asylum except Hanwell in this country. The resident physician scarcely feels himself at liberty to mention other asylums in England and Scotland, in which he knows that the trial is now making under competent superintendents; but he may refer to the liberal manner in which the attempt has been received at Glasgow, at Montrose, at Stafford, and by the directors of the Retreat at York, as well as by the physicians of asylums in America, as encouraging a hope that the number of institutions for the insane in which strait-waistcoats, muffs, restraint-chairs, chains, and straps are no longer seen, will become greater in every succeeding year, until such unnecessary auxiliaries of treatment, if they prove to be unnecessary, are universally abandoned.

"Seclusion.—The resident physician would earnestly impress upon all who may do him the honour to refer to his Reports with a desire either to form a judgment of the plan pursued at Hanwell, or an intention of adopting it, the necessity of considering *all its parts,* of which the discontinuance of mechanical restraints is only one. It is especially necessary to be guarded against the extravagant notions of *seclusion* set forth by the opponents of the non-restraint system. A reference to the Report of last year, in which this very important particular of treatment was fully described, will show, that instead of being an imprisonment productive of every moral and physical evil, it is a simple exclusion from the irritable brain of all external causes of additional irritation; that it places the patient in security, without fretting him by muscular bindings and impediment of limbs; that it secures other patients from danger, and the violent patient from injury when in restraint and defenceless, whilst it removes from his companions a spectacle always displeasing and often alarming to them, that of a patient, who if at large must be degraded by the muff or sleeves; that it very seldom fails to tranquillize the patient in a short time, and is even generally productive of immediate composure; that with all this it is easily effected, whereas the imposition of restraint was often only accomplished after a severe and irritating struggle, and was always most difficult when most required; and, lastly, that it has no general tendency to leave a revengeful feeling in the patient's mind.

"But to secure these advantages of seclusion, it must be remembered that the term is applied to the temporary confinement of a lunatic in his own bed-room; sometimes with the light partially excluded, sometimes almost entirely; that it must not be hastily resorted to; not carried into effect with anger, but steadily accomplished, when persuasion fails, by a sufficient number of attendants; that it must not be accompanied with irritating expressions; nor applied as a punishment; nor unreasonably prolonged. All instances of seclusion should be promptly reported to the medical officer or matron; the state of the patient in seclusion should be ascertained from time to time through the inspection-plate; and any appearance of contrition should be met with kindness. After half an hour, or one hour, or two hours, in different cases, the practicability of putting an end to the seclusion should be tried; except in instances where a longer repose of the brain is plainly required.

"This is the manner in which seclusion is directed to be practised at Hanwell; and although there are many days in the year in which there is not a single patient in seclusion during any part of the day, opportunities of witnessing its remarkable influence in inducing quietness are of course frequently afforded. In conducting visitors through the asylum, their attention is generally directed to the cases actually in seclusion, and whom they are commonly able to observe without occasioning them any disturbance.

"These advantages, there is every reason to think, were never derived from any form of mechanical restraint. The resident physician is aware that an opinion is entertained by a few respectable authorities that restraint exercises a salutary moral effect. He can recall no instance in which it seemed to him to do so. It has also been said that patients accustomed to violent attacks of mania will beg to be put in restraints. He does not disbelieve this; but he has never met with an instance of it. He has occasionally known patients beg that restraints might be put on other patients:

he never knew them ask to have it put on themselves. He has met with one instance in which a patient who recovered some years ago, states that when he was excited he always felt secure as soon as restraints were put on. The same individual, however, is now liable to attacks of excitement; but never solicits to be put in restraint of any kind. Patients liable to attacks of mania in a house where restraints are much employed, regard the accession of an attack with terror; but it is quite possible to screen them from every danger without restraint. The indignation of those on whom restraints were forcibly imposed; their eloquent exposition of its degrading and maddening effects; their fierceness when running about the wards in a strait-waistcoat; the alarm and discontent their presence occasioned to other patients; their speechless joy, their sobs and tears, when unexpectedly liberated, when both in seclusion and restraint, in the midst of a paroxysm of reproaches prolonged until they were covered with perspiration and their lips were literally covered with foam, are things of which the memory can never be effaced from the mind; and they make the ear deaf to specious representations of the comforts of straps and strait-waistcoats. When these results are contrasted with those of simple seclusion; when the angriness, the ferocity, and sullenness characteristic of some of the wards in which restraints were habitually employed (as in the epileptic wards,) are compared with the present indications of confidence and general good temper, the firm impression left is, that the general bad effects of restraint and its liability to abuse are immeasurably more pernicious than anything that can be occasioned by its discontinuance.

"After the trial already made at Hanwell, and a frequent revision of every kind of troublesome case, the resident physician can only concede that there would be some *convenience* in putting certain patients in restraint, who have the inconvenient habit of suddenly striking those about them. But even this convenience would only be temporary, for it cannot be continually applied. Seclusion, therefore, although it does not wholly remove the difficulty of such cases, is a better temporary resource, as it neither leaves the patient offended, nor aggravates his combative propensity, which restraint always seemed to do in exact proportion to its severity and duration. No patients in the asylum were more dangerous to approach than those who were fastened every day in restraint-chairs; and no patients have improved more signally since their entire liberation.

"One cherished error of the advocates of restraint is, that it can at least do no harm to the incurable. The fact is, that no patients are more permanently injured by it, in temper, feelings, and habits. The case of a female patient was last year commented upon as a case in which restraint might have been applied with convenience, as the patient was incurable. This patient (E. W.), after a long and troublesome attack, left the asylum well in July last.

"But, in cases in which there is no hope of cure, the mitigation of violence, ensuing on the long absence of any irritation that can be avoided, is extraordinary. D. E. was admitted in June, 1839. She had been long insane, and was of a violent temper. For some time this patient was daily committing or threatening some violence. Restraint was freely employed: shower-baths and the douche were often resorted to to calm her paroxysms. The baths were apparently useful, but the restraints seemed only to exasperate her the more. At length, every exciting application, mechanical or verbal, being found hurtful, she was spoken to with invariable calmness, and scarcely ever interfered with. She continues liable to violent fits of passion, but seldom treats those about her with incivility, though often desirous of conversing with visitors, and seems to take a pride in conducting herself with a studied and ceremonions politeness.

"A memorandum relative to restraints, furnished by Mrs. Bowden (late Miss Powell,) the matron, comprehends forty-one cases, almost all of which were in *constant* restraint of some kind or other previous to September, 1839. Fourteen of these patients were almost always fastened in restraint-chairs, and twenty were almost always in a kind of strait-waistcoat, called sleeves: several were in complicated restraints, and some in a chair, and at the same time in sleeves, or the muff, or in leg-locks. All these patients were liberated before the end of September, 1839. Not one of them has been in restraint since. Thirty-seven are yet in the asylum, and there is not one who may not be pointed out as an instance of the improvement of the mental faculties, or of the habits, in consequence of never being subjected to restraints during two years. Some, who were considered dangerous at all times, are now occasionally seen at the work-table, smiling, and pointing out what they have done. Some, who were sinking into dementia, or imbecility, are now living and talkative. Some, who were said to pursue visitors through the ward, are now never known to do so. The brief statement of the past and present condition of these patients is one of the most interesting documents ever drawn up in any lunatic asylum, although its preparation is only one of many important services rendered by the matron to the cause of the poor lunatic.

"As at that time no Reports were ever made, or records kept, by the attendants of the patients in restraint, whilst closets full of instruments of restraint were at their command, it was impossible for the resident physician ever to know, by night or by day, how many of the patients whom it was his duty to protect were in actual bondage.

"It was curiously indicative of the perversion of feeling engendered by long familiarity with restraint, that there was no part of the asylum in which they were more freely employed than in the female infirmary; and to one instructive instance of their misapplication the resident physician must ever acknowledge himself indebted, as the cause of his being more immediately roused to forbid practices on which he had always looked with dissatisfaction.

"A young woman (E. D.), in a state of chronic dementia, following attacks of mania which had occurred six or seven years previously, was found, on the resident physician's first visit to the wards after his appointment, fastened in her bed by a strap round her neck, by a strap round her waist, and by straps to her feet. She also had on the sleeves, which enveloped her hands in hard leather cases, and her hands were also fastened by short straps connected with the strap round her waist. She was extremely feeble and emaciated; her skin was in a very irritable state. She could not get out of bed or raise herself up, or turn, or lift her hand to her mouth. In this state she had been kept for some weeks. No cause was assigned except that she was troublesome, that she would undress herself, that she was always in mischief.

"Day by day, with all the caution assumed to be necessary, one after another of her galling restraints was removed. For a short time she proved to be mischievous and troublesome in her powerless way. She delighted in taking off the clothes from her irritable skin, and she preferred standing up to lying down upon the irritating straw of her bed. She one day broke a pane of glass (being still locked up in her room), and squeezed part of her superfluous wardrobe through it. This habit was discontinued when she was permitted to come into the gallery; but, as she was fond of taking any unappropriated bread, tea, or beer that she found in the ward, the infirmary nurse, who had highly disapproved of all the proceedings in the patient's favour, contented herself with the milder means of fastening a long strap to the waist-strap of the patient's dress, and securing her by it to an iron bar, or a bench, or a heavy restraint-chair, in which state visitors shrunk with affright from this poor harmless creature. This thraldom being also forbidden, the patient gradually became less troublesome, and being removed to another ward slowly recovered strength, and even became fat. The poor girl had been a music-mistress, and in a few weeks after her restraints were taken off she was led to the organ in the chapel by the physician's ever kind assistant, the matron, and induced to play. It need scarcely be added that her playing was not listened to without emotion. This patient is yet in the asylum, imbecile, and incapable of employment, but seldom mischievous or troublesome; and so altered are the habits of the attendants, that if she were to be ordered into severe restraint, or into any restraint at all, it would probably create a general feeling of horror and aversion throughout the house.

"The unavoidable impression made by such a case was, that there must be something inherently wrong in a system of treatment, the grossest misapplication of which, witnessed daily by those whose general kindness to the patients was never called in question, appeared to excite no surprise.

"The resident physician is anxious to state the results of the experiment which ensued on these impressions in cautious language, so as to avoid creating unreasonable expectations, but he considers it to be his duty to relate his experience without disguise. He feels able, after two years' careful trial, to state:

"1. That much difficulty will always attend the discontinuance of restraints, in consequence of the indifference and indolence of attendants accustomed to avoid trouble by imposing them on every occasion in which a patient is irritable or mischievous.

"2. That some of the patients who have been long accustomed to severe mechanical restraints must be expected to be troublesome for some time after being restored to freedom of muscular action.

"3. That this disposition may show itself in a proneness to quarrel and fight, or to break windows, or to knock at the door of their bed-rooms during a part of the night. Patients who have been heretofore strapped or chained to the bedstead every night, being liberated, probably fancy it to be day, and knock at the doors that they may be let out. Patients who heretofore passed every day of the year in a restraint-chair have become accustomed to the sole amusement left within their reach, that of striking the passers-by.

"4. That if the attempt be steadily persevered in, the conduct of several of the most troublesome patients will eventually be found to improve, and the attendants will gradually learn that they can manage the patients better without restraint than with it.

"5. That one conspicuous result will be a general tranquillity in wards previously distinguished for excitability and noise. When the epileptic patients have been frequently in restraint, this will be remarkably evident in the epileptic wards. When every epileptic patient is fastened in bed at night, although only by one hand, almost every such patient will be more strictly bound on the approach and for some time after a paroxysm; so that a very great amount of restraint will become habitual in the epileptic wards, and frequent struggles, quarrels, and blows, as well as general discontent and ill humour, will characterize them. The alteration effected by the entire disuse of restraints among them will be proportionably striking, for many of the epileptics are perfectly rational between the paroxysms, and fully appreciate the manner in which they are treated.

"6. That abusive language, assaults, and acts of violence will gradually become of less frequent occurrence.

"7. That a disposition to meditate mischief or revenge will seldom be observed.

"8. That the general aspect of the patients, throughout the house, will become more cheerful and confiding.

"9. That the patients will be quieter at night, apparently enjoying the natural rest that belongs to a state of physical and mental comfort.

"10. That the patients will be less liable to manifest irritation during divine service.

"11. That cases of obstinate refusal to comply with rules and directions, as well as instances of persevering refusal of food, and of attempts to commit suicide, will become less frequent.

"12. That improvement will be more readily effected in patients recently admitted, who will be less alarmed and less excited on admission, and sooner acquire confidence in those about them.

"13. That the abolition of restraints cannot be successful without a vigilant and constant superintendence exercised by a competent number of attendants, under the direction of officers zealously united in the determination to meet every variety of difficulty by every possible resource that may render bodily coercion unnecessary.

"14. That the attendants most attached to the use of restraints will be the least alert in carrying these resources into practice, and the most prone to use secret violence, and to abuse seclusion, until it becomes as objectionable as restraint itself.

"15. That in a properly arranged asylum, with efficient officers and attendants, acting under one uniform system, scarcely any conceivable case can occur in which the security, cleanliness, and comfort of the patient may not be provided for, and the safety of all the other patients secured without mechanical or bodily restraint, and with fewer troublesome consequences than when restraints are employed.

"16. That to effect these objects it is not necessary that the number of attendants should be extravagantly great. At Hanwell it is only in the proportion of one attendant to eighteen patients. But the arrangements must be such that the patients are never left unattended during the day. It is by no means indispensable that the attendants should possess unusual bodily strength. The first requisite is that they should be humane, without which they will never gain the good opinion and confidence of the patients. They must be capable of acting with great patience, and also with firmness and courage. It must be strongly enforced upon them that it is their business to *prevent* mischief, and not to punish it. They must be habituated, when all arts of persuasion fail, and some point of obedience *must* be enforced, to act steadily and systematically together, so as to effect the end with promptness and certainty without provoking words and without a prolonged struggle. Whenever they are required to collect in a certain number, for the purpose of secluding a dangerous patient, they must assemble quickly and quietly, must avoid harassing the patient by controversy and useless contradiction, and, having accomplished the purpose which brought them together, must disperse as quietly as they came. These are directions continually acted upon at Hanwell; and the plan has a considerable effect on the patients, who are perfectly aware that they will not be unnecessarily interfered with, but that when interference is necessary it is sure to be effectual.

"Such are the results of the present experience of the resident physician, gathered in two watchful years, and more strongly confirmed as the experiment has proceeded. He has reason to believe that the experience of other medical observers, residing like himself constantly among the insane, and well acquainted with the state of their particular asylums by night as well as by day, is, as far as it has yet extended, precisely of the same kind.

"General Treatment.—The quality of management which a careful superintendent will be continually learning in a lunatic asylum is *forbearance.* Scenes presenting at first sight an aspect of confusion and violence, are generally found to resolve themselves into simple elements if calmly surveyed for a few minutes. Furious gestures, threatening language, and abusive epithets, if not met by irritable and angry measures, commonly subside in a short time. Determinations of disobedience, apparently the most resolute, often yield to continued and calm persuasion. To all these events there are exceptions; but the exceptions will probably be always the least numerous with superintendents, officers, and attendants who preserve the most command over themselves. Firmness and determination may be even often required, but anger or passion always leave an uncomfortable impression that they have been at least superfluous. In these respects the resident physician has observed a considerable improvement in the ward-attendants since the disuse of restraints. The frequent imposition of hand-locks, or the muff, or the strait-waistcoat, or leg-locks,—the difficulty with which they were put on a violent patient,—the anger this imposition excited against the attendant, especially when put on, as was often the case, needlessly and unjustly, placed many of the patients and attendants in a state of continual hostility to one another. The attendant never spoke well of the patient, and the patient always complained of the attendant. At this time the patients in the refractory male wards are not unfrequently to be seen grouped round an attendant, who plays some instrument for their amusement; and patients, violent at other times, afford essential occasional protection to the attendant, when suddenly taken at disadvantage by some other violent patient. In the female refractory wards, the patients are usually found either assembled round a work-table with the nurses, or sitting by them on benches in the airing court, or riding with them on the rocking horses. In both the male and female wards there is an appearance of a good feeling between the patients and attendants; and when it is necessary to use some force, either to ensure seclusion, or to administer a bath, it is done after long attempts at persuasion, with a quietness, promptness, and efficacy, by which the patient is taken by surprise, and obedience ensured without anything being done which gives the patient much offence.

"Although the number of ward-attendants is much smaller than in some other asylums, in proportion to the number of patients, the general appearance of the wards and of the patients sufficiently attest their efficiency as regards the preservation of cleanliness and order; an effect secured by accumulating attendants in wards containing patients who are violent or dirty. Yet it is to be regretted that the necessity thus created of assigning 50 patients to two attendants in some of the quieter wards should deprive some of them, as it can scarcely fail to do, of attentions to which they would be peculiarly sensible. It is scarcely to be supposed that attendants having the care of so many patients, for whose cleanliness, as well as that of the gallery and numerous bedrooms, they are held entirely responsible, and who have to attend their patients at every meal, can have sufficient energy remaining to employ the intervals between their various household duties, in devising or applying mental or moral resources of treatment to those under their charge.

"A mere change of attendants has heen found, in several instances, to be followed with advantages greater than were expected. The character of a ward seems almost wholly to depend on the character of the attendants. A description of the duties of the ward-attendants has been lately printed in the form of a manual, appended to the report of the visiting justices, in which the duty of every hour of the day, and of every day in the week, is precisely laid down. Strict observance of these rules is expected, and the general regularity of the house is most satisfactory.

"Whatever may be the ability, zeal, or activity of those at the head of an asylum, they must always so much depend on the fidelity and good conduct of the male and female attendants, that it is scarcely possible to be too considerate of whatever tends to keep them in bodily and mental health. Unless they are cheerful and active, and well disposed towards the officers of the asylum, their services to the patients will be inefficiently performed. They are expected to exercise great vigilance for at least fourteen hours every day, and to take their turn of night-watching. No reasonable relaxation, therefore, should be withheld from them; and their lodging, provisions, and every arrangement regarding them, should be calculated to preserve the habits and manners of respectable life, without which they are not likely to understand or execute some of the most important of the duties required of them. The resident physician feels it to be important to allude to this subject, in consequence of having experienced, first, the impossibility of conducting the asylum properly with attendants whose attainments, manners, dress, and language, were those of the lowest class of servants; and, secondly, the facility with which regularity may be maintained, even in a very large asylum, by means of an entirely different class of assistants, encouraged by a more liberal remuneration.

"Religious Services.—Since the appointment of the Reverend J. T. Burt to the office of chaplain, some alteration has been made as regards the daily religious services; the hour of the evening service, except on Sundays, being six o'clock, instead of half-past nine. This change enables many patients to attend every evening, as well as every morning at eight. Each service is commenced by three verses of a hymn or psalm being sung by the congregation, accompanied by the organ. The chaplain has also been kind enough to attend to a suggestion of the physician that an occasional short service should be read to the feeble and infirm patients in the infirmaries who are unable to reach the chapel. The services on Sundays remain unaltered, and take place at eleven and half-past three; each service lasting one hour. In the morning, the prayers of the Church of England are read, and in the afternoon, a portion of the afternoon service, and a sermon. It seldom happens that any irregularity of behaviour occurs among the patients during these services. Most of them observe a demeanour that would become any place of worship; and join in the responses and singing, and pay attention to the sermon. On Sunday, October 10th, the sacrament was administered for the first time since Mr. Burt's appointment, and the number of communicants was sixty, of whom fifty were patients.

"It affords the resident physician much satisfaction to be able to say that the kind and judicious co-operation of the chaplain, and his frequent and unreserved communications, are such as to inspire him with the fullest confidence that the ministerial offices performed in the asylum are carefully adapted to patients so peculiarly afflicted; and administered in a spirit so temperate, earnest, and sincere, as to extend their benefits to every case in which prudence can permit a trial. Long experience can alone determine the cases to which religious attentions are applicable: they are sometimes beneficial where advantage was scarcely hoped for, and sometimes excite when no excitement was expected. They are also not equally applicable to the same patient at all times. Nothing can meet the various difficulties incidental to such delicate trials, except the constant and confidential communications of the physician and chaplain to each other. If the physician may sometimes prevent mischief that might arise from an ill-timed religious appeal, the chaplain is sometimes enabled by his intercourse with the patients to demonstrate that the influence of his spiritual conversation is deeper and more permanent than the physician might expect. By their continual co-operation alone can the zeal, judgment, and experience of both be so combined and directed as to throw light on a subject which, independently of its serious importance in a religious point of view, is connected with the interesting psychological question of how far the feelings and understanding may act and be acted upon in the various shades of mental disorder;—how far, when the mental state disqualifies for worldly duties and pursuits, the affections and the vestiges of reason enable the afflicted lunatic to look up to his Creator. Experiments in relation to this important subject do not yet appear to have been made on a sufficiently extensive scale either to justify enthusiasm or despondency. It is not improbable that future reports may contain some interesting particulars relative to a subject on which at present very little seems to be known.

"Like every other part of a system that appeals to what remains of the reason and the feelings, a perfect trial of what could be effected by spiritual means was incompatible with modes of treatment which produced gloom, discontent, or ferocity. The ordinary public services at Hanwell were much more liable to interruption before the disuse of mechanical restraints; and on looking back to the instances of disturbance which then occurred, it is curious as well as instructive to remember, that the patients who interrupted the services by noise or violence, were almost always such as had been the especial subjects of mechanical restraint. At that time Sunday was a day of more than usual anxiety; whereas there is now no day in which the aspect of the whole asylum is more tranquil and comfortable. The attendants of the patients assemble for chapel neatly dressed; the congregation is usually about 300; and when the services are concluded, the patients may be seen walking down the galleries which lead from the chapel, as orderly as any crowd in a church-way path. Although they have no work to occupy them on Sundays, they may be seen sitting composedly in their wards after the service, or taking their dinners with every appearance of order and comfort. All such general descriptions are liable to occasional exceptions. One noisy patient may disturb fifty patients; and the paroxysms of mania may occur on Sunday as on any other day; but the general character of the house on Sundays is certainly such as is above described.

"The interruptions of illness have during the last year made the resident physician more than usually dependent on the activity and fidelity of the officers and servants of the asylum; and he wishes he could efficiently express his grateful acknowledgments to each and all of them. It is a source of great satisfaction to believe that there is not an officer or attendant in the whole of the numerous establishment who is, at this time, disposed in any way to impede the general and well-known design of the resident physician. From every department,—the medical, the economic, the household, he receives uniform and valuable support.

"Several of the present visiting justices being now about to resign their office, the resident physician hopes he may be permitted to assure them that he shall never cease to reflect with satisfaction on the period when he had the honour, with their support, to adopt, and carry on a system of management which he believes to be founded on correct principles, and to the gradual extension of which, more or less modified, to many or to all other asylums, public or private, he looks forward with confident hope.

"J. CONOLLY."

**[It would be doing great injustice to the Committee of County Magistrates who visit the Asylum, not to state how zealously they have seconded the efforts of Dr. Conolly in carrying out his plans for the regeneration of Hanwell, and—it**

**may be truly said—of lunatic asylums in general. In his own statement, Dr. Conolly gratefully acknowledges their countenance and support; and the following extracts from their "Fifty-ninth Report," in which that of the resident physician is included, prove how well entitled they are to a share of that great honour which the glorious spectacle now exhibited at Hanwell confers on all concerned in its production.]**

*Extracts from the Justices' Report.*

"At the Michaelmas quarter sessions of the last year, a question arose, as to whether the system which had been adopted by the resident physician of the Asylum at Hanwell, and had been sanctioned by the visiting justices, had not been carried out in too rash and unqualified a manner to be practically safe. Such were the conflicting opinions entertained upon this question by intelligent men, that it was then resolved, by the magistrates assembled, to intrust the control of the asylum to the same visiting justices for another year, and to postpone any decision upon the subject, if a decision might be necessary, until the court could have the benefit of the longer experience and the more matured judgment of its committee. It was impossible to doubt the propriety of the course which was then resolved upon, and the visiting justices felt it to be their duty to enter once more upon the trust which was confided to them, of watching, with calm and unprejudiced minds, the practical working of the system; prepared, on the one hand, to give it their steady support, should the further examination of the facts which might come under their notice bear out their favorable opinions, or, on the other hand, so to modify it, as to remove the reasonable objections of those who deemed it to be both theoretical and dangerous.

"The points upon which the visiting justices have specially to report are, whether, in the management of the patients at Hanwell, the mechanical means heretofore employed in restraining violence, or in preventing accidents, can be altogether dispensed with without great danger to the insane or to their attendants; and whether there may not be more of actual cruelty hidden under the show of humanity in the system of non-coercion, than was openly displayed in muffs, strait-waistcoats, leg-locks, and coercion chairs. Both of these are very serious and important questions to answer, the one involving the personal safety of the patients, and the other their personal comfort, which all along the committee had been studiously endeavouring to promote.

"It is quite clear that had the choice been between constant personal restraint as a preventive against accidents or dangerous violence, and no restraint at all, the question might easily be answered. It belongs especially to the malady with which the insane are afflicted, that they are uncertain and capricious in their actions; that they are liable, with little or no apparent cause, to paroxysms of fury, that there are strong suicidal tendencies to be found among them, and in the cases of the idiotic and the epileptic, that their very helplessness is frequently a source of danger. Against these it is impossible at all times to guard so effectually as to afford absolute and perfect security, except it be by the application of the most rigorous mechanical restraint, and that without intermission. Every relaxation of this mode of management, must, from the very uncertain nature of the disease, be attended with some degree of danger. This is inevitable, and if every other consideration of a far higher nature must be sacrificed to perfect security from all danger, such selfish views can only be realized by reverting to the practice of past times and of a barbarous people, when the insane were chained and beaten as if they were wild animals, who had nothing of humanity remaining but the outward form; and when the idiotic from whom nothing was to be feared, were left to roam at large uncared for, and unprotected. But the question is the comparative security of two plans, both of them necessarily imperfect; the one, imperfect from the occasional intermission of mechanical coercion, at times possibly when it might be most required, and the other, from depending chiefly upon moral means for security, and not at all upon instrumental restraints. That the former plan offers but a very imperfect safeguard against dangers of every kind is put beyond a doubt, by the instances of suicide, and attempts at suicide, of violence, attended with fatal or serious consequences, and of accidents, which are to be found in the records of every institution, including from its commencement those of the asylum at Hanwell.

"But do the means which are now adopted at Hanwell offer an equal security against the dangers to which every institution, and especially every large institution for the insane is liable? Are they sufficient to guard, not with absolute certainty, but with reasonable hope against danger, equal at least to the mode of management for which it has been substituted? The visiting justices beg to answer distinctly in the affirmative; and to state, as the result of their experience, that notwithstanding the many obstacles with which the non-restraint system has had to struggle, notwithstanding the difficulties which have unavoidably attended the transition from one system to another, notwithstanding all these, and even more than these obstacles by which the means have been materially weakened that were relied on for complete success, that success has fully equalled their hopes, and has presented advantages, which in their opinion more than compensate the imperfections to which every human contrivance is liable.

"The committee conceive it must be obvious to every one, that whatever means can be applied to cheer and divert the minds of the patients, must at the same time tend to awaken their susceptibility to kindness, increase the moral control of the officers who contribute to their enjoyment, and in the same proportion diminish the necessity of governing them by brute force and by mechanical restraints. In fact, no such necessity has arisen in any one instance during the present year, any more than during that which is past, and it is not unreasonble to ascribe it to these among other means which have been employed in bringing about so desirable and, to some, so unlooked-for a result.

"And yet it has been supposed that there is actually more of cruelty in this system, under the guise of humanity, than in that which was partly carried on by instruments of coercion! To this the visiting justices would beg in reply to say, that they can detect no grounds for so serious an imputation. If it had altogether failed, and by its failure entailed unnecessary sufferings upon the patient, then indeed it might possibly be open to such a charge. But such is not the case. It has not failed, but has succeeded; and the visiting justices have perhaps one of the best proofs of its success in the testimonies of the patients themselves when they are restored to health, and are fully alive to the comforts and advantages which they have enjoyed under it. The committee are accustomed, on their appearing before them to receive their discharge, to examine them as to the degree of consciousness they possessed during their malady, and it has been found that their consciousness, and their recollection of what had passed, were much more distinct and perfect than might be supposed. In answer to enquiries as to whether they were satisfied with their treatment, and whether they had anything to complain of, they have uniformly, with but one exception, where a female complained of having been once struck on the face by a nurse, expressed themselves perfectly satisfied with the kindness and attention they had received, and in some instances in such feeling terms as to convince the visiting justices that kind treatment is not lost upon the insane, but is distinctly and gratefully recollected when they are restored to reason.

"Strengthened in their deliberate judgment by men of great eminence in the profession, both natives and foreigners, and by the example of other public institutions, and especially of the large asylum for the insane in the county of Lancaster, which contains between five and six hundred patients, and in which, under the direction of Dr. de Vitre, the physician, and of Mr. Gaskill, the surgeon and superintendent, 'watchfulness, care, and ingenuity,' in the words of their report for this year, 'are now employed as substitutes for the instruments of coercion;' strengthened by these, and by the approach to a better system which is at this time being gradually made in several of our great establishments for the insane, the visiting justices conceive that their experience fully authorizes them in reporting thus favorably of the system to the court, and in earnestly recommending its continuance to their successors.

"CHAS. AUG. TULK, Chairman."

